# Incidence of Major Depressive Disorder: Variation by Age and Sex in Low-Income Individuals: A Population-Based 10-Year Follow-Up Study

**DOI:** 10.1097/MD.0000000000003110

**Published:** 2016-04-18

**Authors:** Chun-Te Lee, Yi-Cheng Chiang, Jing-Yang Huang, Disline M. Tantoh, Oswald N. Nfor, Jia-Fu Lee, Cheng-Chen Chang, Yung-Po Liaw

**Affiliations:** From the Department of Psychiatry (C-TL); School of Medicine (C-TL); Department of Public Health and Institute of Public Health (Y-CC, J-YH, DMT, ONN, Y-PL); Department of Family and Community Medicine (Y-PL), Chung Shan Medical University Hospital, Taichung; Department of Psychiatry (J-FL), Taipei Tzu Chi Hospital, Buddhist Tzu Chi Medical Foundation, Taipei; School of Medicine (J-FL), Tzu Chi University, Hualien; Institute of Medicine (C-CC), Chung Shan Medical University, Taichung; and Department of Psychiatry (C-CC), Changhua Christian Hospital, Changhua, Taiwan.

## Abstract

Major depressive disorder (MDD), the most prevalent mental disorder is a global public health issue.

The aim of this study was to assess the association between low income and major depressive disorder (MDD) by age and sex.

The National Health Insurance Research Database (NHIRD) of Taiwan was used to retrieve data. A total of 1,743,948 participants were eligible for the study. Low-income individuals were identified from 2001 and 2003 (specifically, Group Insurance Applicants, ie, category“51” or “52”) and followed from 2004 to 2010. MDD was identified using the *ICD-9-CM* 296.2 and 296.3 codes.

Among non-low-income individuals, the MDD incidence rates increased with age in both males and females, that is, 0.35, 0.93, 0.97, 1.40 per 10,000 person-months for males and 0.41, 1.60, 1.89, 1.95 per 10,000 person-months for females aged 0 to 17, 18 to 44, 45 to 64, and ≥65 years, respectively. Low-income females (18–44 years) and males (45–64 years) had the highest incidence of MDD, which was 3.90 and 3.04, respectively, per 10,000 person-months. Among low and non-low-income individuals, the MDD incidence rates were higher in the females than males in all age groups. Males aged 45 to 64 and 0 to 17 years had highest hazard ratios (HR) of 2.789 (95% confidence interval [CI], 1.937–4.014) and 2.446 (95% CI, 1.603–3.732), respectively. The highest HRs for females were 2.663 (95% CI, 1.878–3.775) and 2.219 (CI, 1.821–2.705) in the 0 to 17 and 18- to 44-year age groups. Low income was not found to serve as a risk factor for the development of MDD in males and females aged ≥65 years.

Among the non-low-income males and females, the incidence rates of MDD were found to increase with age. Low income was found to serve as a significant risk factor for MDD only in individuals under age 65.

## INTRODUCTION

Depression is an important public health issue.^1^ Globally, major depressive disorder (MDD) is the most prevalent mental disorder which is often long-lived, causing substantial impairment.^2–4^ In 1996, the prevalence of MDD in Taiwan and Korea was 0.8% and 2.3%, respectively, whereas that in western countries ranged from 3.0% in the United States to 5.8% in New Zealand.^5^ A cross-national study carried out in 10 countries across America, Europe, and Asia in 2003 estimated lifetime prevalence of MDD, which ranged from 3.0% in Japan (Asia) to 16.9% in the United States (North America).^6^ Several cross-sectional studies have associated low income with MDD.^7–9^ For instance, low levels of household income were associated with a number of lifetime mental disorders.^10^ Furthermore, a large-scale, cross-sectional epidemiological study on MDD among urban and rural residents in Beijing found low family income and female sex, among others, as independent risk factors of MDD.^11^ However, large-scale cohort studies on the relationship between low income and MDD have rarely been conducted. Therefore, this study aimed at conducting a population-based 10-year follow-up study to explore the association between low income and MDD by age and sex.

## MATERIALS AND METHODS

The primary data source was the National Health Insurance Research Database (NHIRD). Participants’ informations were obtained from 2001 to 2010 using the Longitudinal Health Insurance Databases (LHID 2005 and 2010), each comprising 1 million individuals. The NHIRD contains detailed healthcare data of >25 million enrollees, representing >99% of Taiwan's population. Low-income individuals (specifically, Group Insurance Applicants, ie, a category “51” or “52”) were identified from 2001 and 2003.

All inpatients and outpatients with depressive disorders were identified using the International Classification of Diseases, Ninth Revision, Clinical Modification (ICD-9-CM) codes. Specifically, codes 296.2 and 296.3 were used to identify MDD. Individuals diagnosed with MDD from 2001 to 2003 were excluded from the analysis. Also excluded were 20,689 individuals whose income status changed from non-low to low from 2004 to 2010. The Institutional Review Board of Chung Shan Medical University Hospital, Taichung City, Taiwan, approved this study.

*χ*^2^ was used to analyze nominal variables and to compare the proportion of low and non-low-income individuals. Cox proportional hazard model was used to investigate the hazard ratio (HR) of MDD in low-income individuals. Adjustments were made for age, geographic location, urbanization level, and Charlson comorbidity index. The proportional assumption of Cox regression models was tested. Statistical analyses were performed using the SAS 9.3 statistical software (SAS Institute, Cary, NC).

## RESULTS

The demographic characteristics of low-income and non-low-income individuals are shown in Table 1. Eligible for the study were 15,417 low-income individuals (7247 males and 8170 females) and 1,728,531 non-low-income individuals (854,106 males and 874,425 females) from 2001 to 2003. The proportion of low-income females (52.99%) was significantly higher than their non-low-income counterparts (50.59%). However, the proportion of non-low-income males (49.41%) was significantly higher than their low-income counterparts (47.01%). Among the various age groups, the proportions of low- and high-income individuals were significantly different. The proportion of non-low-income individuals aged 18 to 44 years was significantly higher than that of other age groups. However, the proportion of low-income individuals aged 0 to 17 years (43.85%) was significantly higher when compared with other age groups. Considering the geographical location, urbanization level, and Charlson comorbidity index, the proportion of low-income individuals differed significantly when compared with their non-low-income counterparts. From 2004 to 2008, the proportion of low-income individuals diagnosed with MDD (1.73%) was significantly higher than non-low-income individuals (0.92%).

**TABLE 1:**
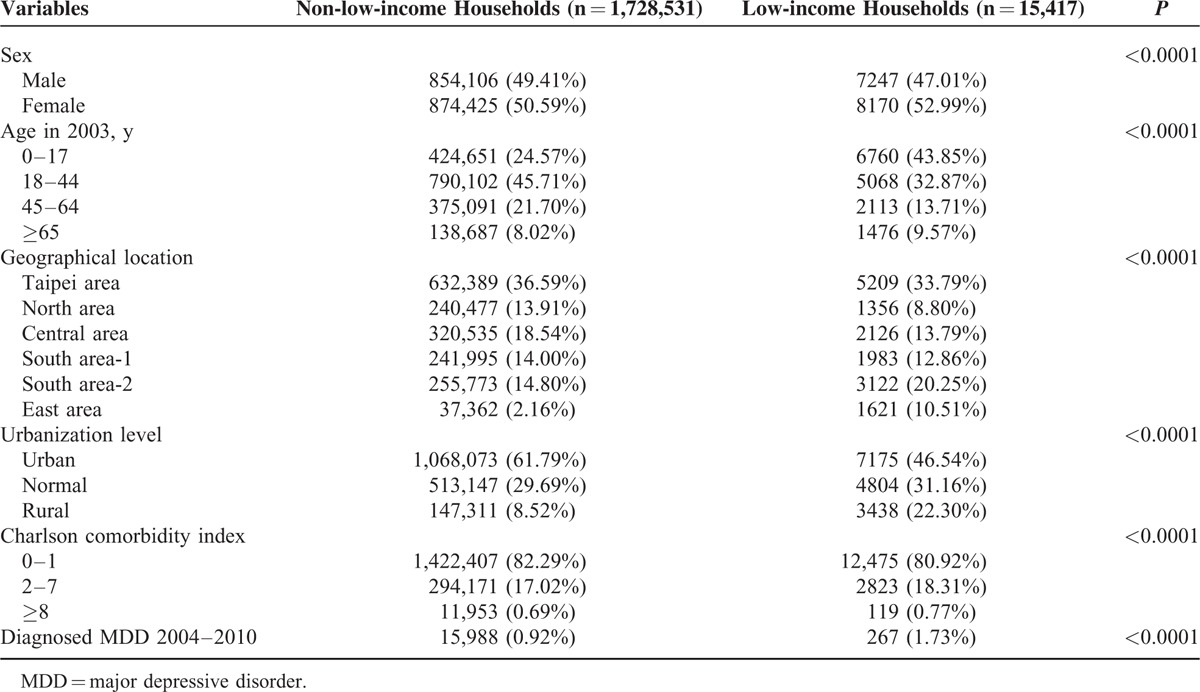
Demographic Characteristics of Low and Non-low-income Individuals From 2001 to 2003 (n = 1,743,948)

Table 2 shows the incidence rates of MDD in the non-low-income and low-income patients categorized by age and sex. Among non-low-income individuals, the MDD incidence rates increased with age in both sexes. That is, 0.35, 0.93, 0.97, 1.40 per 10,000 person-months in males and 0.41, 1.60, 1.89, 1.95 per 10,000 person-months in females aged 0 to 17, 18 to 44, 45 to 64, and ≥65 years, respectively. Low-income males aged 45 to 64 years had the highest MDD incidence rate (3.04 per 10,000 person-months), followed by males aged 18 to 44 years (1.99 per 10,000 person-months), ≥65 years (1.59 per 10,000 person-months), and 0 to 17 years (0.86 per 10,000 person-months). Nonetheless, low-income females aged 18 to 44 years had the highest MDD incidence rate (3.90 per 10,000 person-months) followed by those aged 45 to 64 years (3.77 per 10, 000 person-months), ≥65 years (1.75 per 10,000 person-months) and 0 to 17 years (1.17 per 10,000 person-months). In both low- and non-low-income individuals, the MDD incidence rates were higher in females than males of all the age groups. Among non-low-income individuals, the female to male MDD incidence ratio (1.95) was highest among the age group 45 to 64 years. Among the low-income individuals, the incidence ratio was highest (1.96) among those who were 18 to 44 years old.

**TABLE 2:**
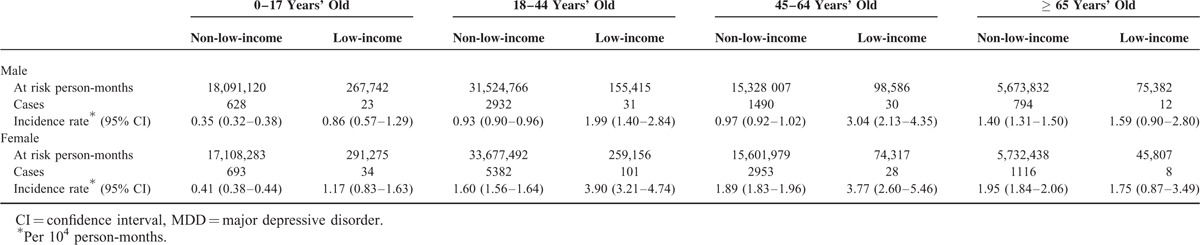
Incidence Rate of MDD Among Low and Non-low-income Individuals From 2004 to 2010

The proportional assumption of Cox regression models was tested. However, the output was not significant (*P* = 0.6987). This indicates that the effect of low income on MDD was constant over time; hence, the proportional assumption of Cox regression models was met. Table 3 shows the HRs for MDD in low-income males and females in various age groups. Men aged 45–64 and 0–17 years were noted with the highest HRs of 2.789 (95% confidence interval [CI], 1.937–4.014) and 2.446 (95% CI, 1.603–3.732), respectively. Among females, the HR of MDD was found to decrease with age, that is, 2.663 (95% CI, 1.878–3.775), 2.219 (95% CI, 1.821–2.705), 1.902 (95% CI, 1.311–2.761), and 0.883 (95% CI, 0.440–1.771) among 0 to 17, 18 to 44, 45 to 64, and ≥65 years age groups, respectively. Low income was not found to serve as a risk factor of MDD in both males and females aged ≥65 years.

**TABLE 3:**
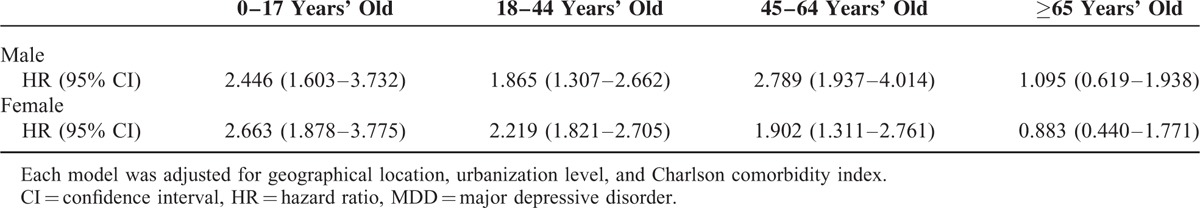
MDD Hazard Ratios Among Low-income Patients

## DISCUSSION AND CONCLUSIONS

This is the first large-scale longitudinal follow-up study to evaluate the association between low income and MDD by age and sex. In this study, income was significantly associated with MDD as opposed to some previous studies. A cross-sectional study of relatively smaller sample size (clinical depression n = 330) reported higher MDD prevalence in individuals with lower monthly income. However, income was not found to serve as a significant risk factor.^12^ Moreover, in this study, the incidence but not prevalence rate was determined. In another cross-sectional study on major depression during the economic crisis in Greece, higher numbers of depressive symptoms were found in more financially strained than less-strained individuals.^7^ Nonetheless, our study found an association between low income and MDD by sex and age.

Liu et al^11^ found that female sex, age above 45 years, low family income, and poor family environment were independent risk factors of MDD. However, the study was not stratified by income. Interestingly, this study had more findings. For example, among low and non-low-income individuals, the MDD incidence rates were higher in females than males in all age groups. Furthermore, low income was not found to serve as a risk factor of MDD in both males and females aged ≥65 years. A previous study found age ≥64 years and low income among others as independent risk factors of MDD in diabetes patients.^13^ The finding that older individuals (≥65 years’ old) are not associated with depression is not surprising. This is because older adults have been shown to be more protective against depression,^14^ as they seem to react less to stressors than younger adults.^15^ Another finding of this study was that low-income females aged 18 to 44 years had the highest incidence of MDD, whereas those aged 0 to 17 years had the highest HR for MDD. However, in low-income males, the highest incidence rate and HR of MDD were found in individuals aged 45 to 64 years. A study conducted on Chinese adolescents showed that girls had more depressive symptoms than boys.^16^ These results align with our findings wherein low-income females between the ages of 0 and 17 years had higher incidence rates and HR of MDD than males. Some possible explanations for these findings are girls have been found to experience higher levels of interpersonal stress than boys, and tend to show more anxiety and depression when confronted with interpersonal stress in the family and among peers.^17,18^ The differences in depression between males and females escalate from early to late adolescence probably because girls are more likely to blame themselves for relationship problems and are more concerned about negative evaluations by peers than boys. Besides, once distressed, adolescent girls may even continue to create a more stressful environment.^17^ A study conducted on adolescent girls associated body dissatisfaction and body mass index with depression.^19,20^ Adolescence is a time of increased vulnerability to depression. Females who may be aware of their low-income status at that stage are likely to be more predisposed to depression.

Some studies showed that women were roughly twice as likely as men to be classified as having major depression.^21–24^ In such studies, however, subjects were not grouped by low income and age as in the present study. The female to male MDD incidence ratios among non-low-income individuals aged 0 to 17, 18 to 44, 45 to 64 and ≥65 years were, respectively, 1.17, 1.72, 1.95, and 1.39. Among low-income individuals, the female to male MDD incidence ratios were 1.36, 1.96, 1.24, and 1.10 for the age groups 0 to 17, 18 to 44, 45 to 64, and ≥65 years, respectively. These variations observed between females and males may be related to income and age.

In this study, we assessed the proportional assumption of the Cox regression models. The output was not significant (*P* = 0.6987). This is an indication that the effect of low income on MDD was constant over time; hence, the proportional assumption was met. Additionally, the KM-plots stratified by age and sex showed that the curves did not cross or intersect each other (figures not shown).

It is possible that the effects of income on MDD may vary over time. However, there were no data on the time-varying income variable; hence, the effects of varying income on MDD over time could not be determined. Individuals were simply divided into low and non-low-income groups. Those whose status changed from non-low to low income from 2004 to 2010 were excluded to minimize potential bias. That is, participants in the control group were those with stable income.

There were some major confounding factors that were not considered during analysis. The association between low income and MDD may have been confounded by environmental (including policy or social support system) and physiological problems (such as depression, anxiety disorders, schizophrenia, personality disorders, adjustment disorders, and family problems). However, these variables were not available and thus could not be adjusted for. This was our study limitation.

In conclusion, MDD incidence rates for the non-low-income men and women were found to increase with age. Furthermore, low-income females aged 18 to 44 years and males aged 45 to 64 years had the highest incidence of MDD. Finally, low income was found to serve as a significant risk factor of MDD only in individuals younger than 65 years.

## References

[1] Chang HY Shin Y-J Batty GD Measuring depression in South Korea: validity and reliability of a brief questionnaire in the Korean Cancer Prevention Study. *J Affect Disord* 2013; 150:760–765. 23541487 10.1016/j.jad.2013.02.035

[2] Demyttenaere K Bruffaerts R Posada-Villa J Prevalence, severity, and unmet need for treatment of mental disorders in the World Health Organization World Mental Health Surveys. *JAMA* 2004; 291:2581–2590. 15173149 10.1001/jama.291.21.2581

[3] González HM Tarraf W Whitfield KE The epidemiology of major depression and ethnicity in the United States. *J Psychiat Res* 2010; 44:1043–1051. 20537350 10.1016/j.jpsychires.2010.03.017 PMC2963677

[4] Kessler RC Birnbaum HG Shahly V Age differences in the prevalence and co-morbidity of DSM-IV major depressive episodes: results from the WHO World Mental Health Survey Initiative. *Depress Anxiety* 2010; 27:351–364. 20037917 10.1002/da.20634 PMC3139270

[5] Weissman MM Bland RC Canino GJ Cross-national epidemiology of major depression and bipolar disorder. *JAMA* 1996; 276:293–299. 8656541

[6] Andrade L Caraveo-anduaga JJ Berglund P The epidemiology of major depressive episodes: results from the International Consortium of Psychiatric Epidemiology (ICPE) Surveys. *Int J Methods Psychiatr Res* 2003; 12:3–21. 12830306 10.1002/mpr.138 PMC6878531

[7] Economou M Madianos M Peppou LE Major depression in the era of economic crisis: a replication of a cross-sectional study across Greece. *J Affect Disord* 2013; 145:308–314. 22939388 10.1016/j.jad.2012.08.008

[8] Butterworth P Rodgers B Windsor TD Financial hardship, socio-economic position and depression: results from the PATH Through Life Survey. *Soc Sci Med* 2009; 69:229–237. 19501441 10.1016/j.socscimed.2009.05.008

[9] Lewis G Bebbington P Brugha T Socio-economic status, standard of living, and neurotic disorder. *Int Rev Psychiatry* 2003; 15:91–96. 12745315 10.1080/0954026021000045994

[10] Sareen J Afifi TO McMillan KA Relationship between household income and mental disorders: findings from a population-based longitudinal study. *Arch Gen Psychiatry* 2011; 68:419–427. 21464366 10.1001/archgenpsychiatry.2011.15

[11] Liu J Yan F Ma X Prevalence of major depressive disorder and socio-demographic correlates: Results of a representative household epidemiological survey in Beijing, China. *J Affect Disord* 2015; 179:74–81. 25845752 10.1016/j.jad.2015.03.009 PMC7127303

[12] Topuzoğlu A Binbay T Ulaş H The epidemiology of major depressive disorder and subthreshold depression in Izmir, Turkey: Prevalence, socioeconomic differences, impairment and help-seeking. *J Affect Disord* 2015; 181:78–86. 25933098 10.1016/j.jad.2015.04.017

[13] Egede LE Zheng D Independent factors associated with major depressive disorder in a national sample of individuals with diabetes. *Diabetes Care* 2003; 26:104–111. 12502665 10.2337/diacare.26.1.104

[14] Fiske A Wetherell JL Gatz M Depression in older adults. *Annu Rev Clin Psychol* 2009; 5:363. 19327033 10.1146/annurev.clinpsy.032408.153621 PMC2852580

[15] Neupert SD Almeida DM Charles ST Age differences in reactivity to daily stressors: The role of personal control. *J Gerontol B Psychol Sci Soc Sci* 2007; 62:216–225. 10.1093/geronb/62.4.p216 17673531

[16] Xie B Reynolds K Palmer PH Longitudinal analysis of weight perception and psychological factors in Chinese adolescents. *Am J Health Behav* 2011; 35:92. 20950162 10.5993/ajhb.35.1.9 PMC2957668

[17] Rudolph KD Gender differences in emotional responses to interpersonal stress during adolescence. *Adoles Health* 2002; 30:3–13. 10.1016/s1054-139x(01)00383-4 11943569

[18] Hampel P Petermann F Perceived stress, coping, and adjustment in adolescents. *J Adoles Health* 2006; 38:409–415. 10.1016/j.jadohealth.2005.02.014 16549302

[19] Rawana JS The relative importance of body change strategies, weight perception, perceived social support, and self-esteem on adolescent depressive symptoms: Longitudinal findings from a national sample. *J Psychosom Res* 2013; 75:49–54. 23751238 10.1016/j.jpsychores.2013.04.012

[20] Schulte SJ Thomas J Relationship between eating pathology, body dissatisfaction and depressive symptoms among male and female adolescents in the United Arab Emirates. *Eat Behav* 2013; 14:157–160. 23557812 10.1016/j.eatbeh.2013.01.015

[21] Oh DH Kim SA Lee HY Prevalence and correlates of depressive symptoms in korean adults: results of a 2009 korean community health survey. *J Korean Med Sci* 2013; 28:128–135. 23341723 10.3346/jkms.2013.28.1.128 PMC3546091

[22] Van de Velde S Bracke P Levecque K Gender differences in depression in 23 European countries. Cross-national variation in the gender gap in depression. *Soc Sci Med* 2010; 71:305–313. 20483518 10.1016/j.socscimed.2010.03.035

[23] Kessler RC Bromet EJ The epidemiology of depression across cultures. *Annu Rev Public Health* 2013; 34:119. 23514317 10.1146/annurev-publhealth-031912-114409 PMC4100461

[24] Nolen-Hoeksema S Gender differences in depression. *Curr Dir Psychol Sci* 2001; 10:173–176.

