# Incidence of major depressive disorder: variation by age and sex in low-income individuals: a population-based 10-year follow-up study: Erratum

**DOI:** 10.1097/MD.0000000000009525

**Published:** 2017-12-29

In the article, “Incidence of Major Depressive Disorder: Variation by Age and Sex in Low-Income Individuals: A Population-Based 10-Year Follow-Up Study”,^[1]^ which appeared in Volume 95, Issue 15 of *Medicine*, the affiliation for Dr. Chun-Te Lee should appear as “From the Department of Psychiatry (C-TL), Chung Shan Medical University Hospital, Taichung; School of Medicine (C-TL), Chung Shan Medical University, Taichung.”
